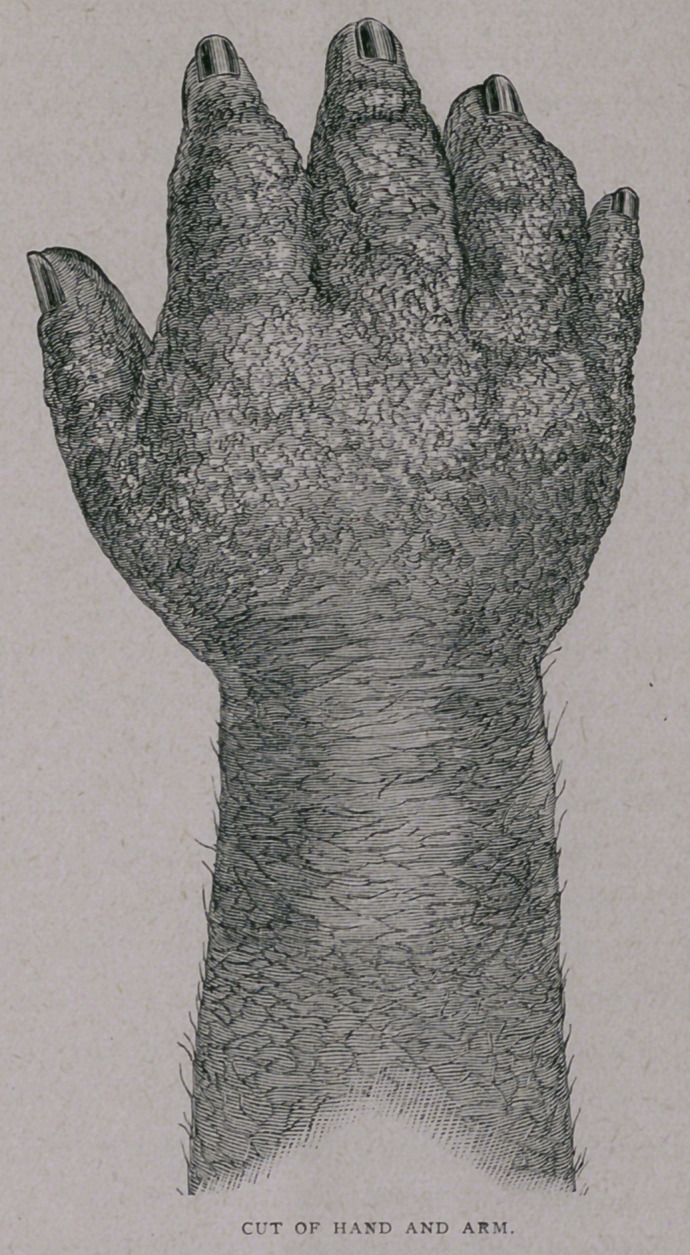# Elephantiasis of the Hand—Amputation—Recovery

**Published:** 1886-05

**Authors:** B. P. Hoyer

**Affiliations:** Physician to the Erie County Penitentiary, Buffalo, N. Y.


### Elephantiasis of the Hand—Amputation—Recovery.

By B. P. Hoyer, M. D.

Physician to the Erie County Penitentiary, Buffalo, N. Y.

Elephantiasis is one of the rarest of lesions observed by American physicians. A few cases are occasionally reported in our medical journals, and are looked upon as curiosities. In India and some other tropical countries, however, the disease is

CUT OF HAND AND ARM.

not rare. Hirsch, in his *Pathologie Hist.-Geograph.*, mentions a district in India where, among 486,000 inhabitants, 2,133 individuals, or 4½ per cent. of the population, were affected with elephantiasis. In the great majority of cases, the lower extremities, or genitalia, are affected. Many writers describe only two varieties of the disease—elephantiasis cruris and elephantiasis genitalium. On the other hand, Rokitansky, Kaposi and Hebra have described elephantiatic enlargement in other locations—the cheek, nose, hand and arm. In these cases, where a great number of blood vessels have been observed in the swelling, the disease has been denominated telangiectatic elephantiasis.

In these cases, according to Geber, the *filaria sanguinis* is not necessarily present. The diagnosis is made (1) from the great hypertrophy of the tissues; (2) diminution or destruction of sensation and motion; (3) pale, shiny appearance of skin, with exudation of milky lymph; (4) the clinical history—the disease is usually preceded by nervous disturbances, followed by erysipeloid inflammation and progressive enlargement of the superficial tissues, with marked changes in the lymphatic system; (5) in tropical climates the filaria sanguinis hominis is usually found, but that this is the case in temperate regions is by no means demonstrated.

In regard to treatment, amputation or excision were among the earliest methods adopted in the East. When the genitalia were affected, a complete cure was often obtained, but the result was generally unfavorable when the leg was amputated. Some died of pyæmia, others survived the operation, but there was a recurrence of the disease. Surgeons who believed the hypertrophy to be due to an excessive blood supply have ligated the femoral or iliac artery.

Of 69 cases treated in this way, 40 recovered, thirteen improved, and the condition remained unchanged in 16. Others regarding the affection as a nutritive disturbance have attacked the sciatic nerve instead of the blood vessels.

Martin reported a case successfully treated in this manner.

Muncorvo, of Rio Janeiro, obtained some success in the treatment of these cases by electrolysis.

The case which we are about to report presents all the characteristic symptoms of elephantiasis, excepting the presence of the embryonal filaria in the blood. Repeated searches were made for these by competent microscopists, but always with a negative result.

Charles F. A., aged 26, of English parentage, entered the Erie County Penitentiary June 25, 1885. About one month after his admission, there was a sudden loss of sensation in the right hand, followed by motor disturbances and enlargement. His condition steadily grew worse until December.

At this time erysipelas, involving the hand and forearm, occurred, lasting about a week. As the erysipelas subsided there was a slight increase of sensation and motion. The hypertrophy, however, increased, and sensation again disappeared. Jan. 1, 1886, the patient began to have epileptiform convulsions, lasting from five to eight minutes, and attended with tonic spasms of the legs and arms. The convulsions usually appeared about sunset, occurring at intervals during several hours. Bromide and other antispasmodics were given to ward off these attacks, but without any apparent beneficial result, for the convulsions only increased in frequency and severity. The knee-joint became affected. For many days all power of motion was lost, although there was no inflammation or swelling. The hand was now enormously enlarged, the integument presenting a shining white appearance, from which a milky lymph at times exuded, and the structural changes were so great that amputation seemed to be the only feasible means of arresting the progress of the disease. The hand had become a burden to its owner, and he earnestly requested that the operation be performed. Drs. Park and Tremaine were consulted, and considered the operation proposed perfectly justifiable, since all the ordinary therapeutic measures had been tried in vain. Antisyphilitic treatment, arsenic, tonics, bandaging and electrolysis had all been equally unsatisfactory, and it was deemed too late to attempt nerve stretching or neurotomy, as advocated by some authors, or ligation of the brachial artery, since this would almost necessarily produce sloughing and gangrene.

On Feb. 22d, with the assistance of Drs. Campbell, Clark, Thornton and Baker, I amputated the arm at the insertion of the deltoid muscle. Esmarch's bandage was used, and circular operation adopted. No difficulties were encountered until after having ligated the large arteries, usually tied in this amputation; the rubber bandage was relaxed.

All present were now astonished by the large number of vessels spouting bright arterial blood. Twenty-six arteries, varying in size from that of a knitting-needle to that of the brachial, required ligation, besides many others from which the hemorrhage was arrested by torsion. There was a remarkable flow of blood from the medullary cavity of the humerus; this was arrested by the application of dry persulphate of iron. After the hemorrhage was entirely controlled, the wound was dressed antiseptically.

About 1 P. M. the patient was put into bed, and morphine, gr. $\frac{1}{4}$, administered hypodermically. He rested during the afternoon, and in the evening no evidence of shock was observed. On the following day, however, there was a rise of temperature, which continued for several days, as may be observed from the following table:

| Date. | Morning Temp. | Morning Pulse. | Evening Temp. | Evening Pulse. | Remarks. |
|---|---|---|---|---|---|
| Feb. 22 | | | $98\frac{3}{5}$ | 74 | 8 P. M. |
| " 23 | $99\frac{2}{5}$ | 82 | $102\frac{2}{5}$ | 106 | |
| " 24 | $101\frac{1}{2}$ | 100 | $102\frac{4}{5}$ | 120 | Maximum Pulse and Temperature. |
| " 25 | 101 | 96 | 102 | 98 | |
| " 26 | $100\frac{1}{5}$ | 90 | $101\frac{1}{5}$ | 90 | |
| " 27 | $100\frac{3}{5}$ | 82 | $99\frac{3}{5}$ | 80 | Wound dressed. |
| " 28 | $98\frac{1}{2}$ | 72 | $99\frac{1}{2}$ | 85 | |

On the second day after the operation the pulse and temperature reached their maximum, and epistaxis, lasting a half hour, occured, with the loss of about two ounces of blood. A small dose of quinine was given, and the temperature was reduced.

Four days after the operation the wound was dressed, considerable pus was evacuated, and some of the stitches removed. With the exception of the accumulation of pus, the wound appeared in a healthy condition. After the dressing the temperature and pulse became nearly normal nor was there, subsequently, any appreciable rise.

Ten days after the operation, patient had a slight convulsion, followed by hemorrhage from the stump; the wound broke open, and it became evident that union by first intention was impossible. The hemorrhage was readily controlled by pressure, and the wound healed by granulation.

Immediately after the operation the stiffness of the knee-joint disappeared, and he has had no nervous disturbances since his recovery. At the present date, April 20th, there is no indication of a recurrence of the hypertrophy.

As some doubt regarding the diagnosis was expressed by those not familiar with the case, I sent the specimen to Professor Arthur Van Harlingen, the well-known dermatologist, of Philadelphia. The following letter, received from him, we give in full :

APRIL 14, 1886.

"DR. B. P. HOYER:

"*Dear Doctor*—I think I told you in the note I sent back with your specimen that the case was undoubtedly one of elephantiasis. It presented all the gross characteristics of that disease, and, so far as it could be examined microscopically, the minute features also.

"My friend, Dr. Ralph W. Seiss, of this city, a well-known microscopist and histologist, has made some sections and examined them, and I give his report:

'*Dear Doctor*—I have carefully examined a number of sections made from the piece of hypertrophied skin which you submitted to me for examination. It is nearly impossible to make an accurate report on the specimens, the deeper layers being much hidden and distorted by post-mortem changes.

'The horny layer is increased in thickness, and its lower layers made up of unusually dense connective tissue. The sulci are also deepened. The deeper layers, so far as may be studied in the degenerated areas, are everywhere densely infiltrated with embryonal small cells, which, in many places, are organized, or organizing, into fibrous tissue.

'The inner stratum is largely infiltrated with inflammatory round cells.

'The lesions shown are simply those of chronic hypertrophic dermatitis or cellulitis, the inflammation being of a plastic character. RALPH W. SEISS.'

"I may add that we found, on opening the hand, that the preservative fluid had only penetrated a short distance beneath the surface, so that the deeper layers were more or less decomposed. This prevented a study of the lymphatic changes, which would have been very interesting.

"I am glad you intend publishing the case, which is of high interest, and I hope you will be able to give full details of the clinical history.

"Cases of elephantiasis of the upper extremity are very rare. I have notes of one case which I saw some years ago with Professor Duhring, but it has not, I believe, ever been published.

"Dr. George H. Fox, of New York, in giving a brief account of a case coming under his observation (*Jour. Cutaneous and Venereal Dis.*, May, 1885, p. 144), says it is the only case he has ever seen, and the only one, perhaps, which has ever been observed in this country (?). Waring, quoted by Tilbury Fox (*Skin Diseases, 2d American Ed.*, p. 361), observed but four cases of elephantiasis of the upper extremity in nine hundred and forty-five.

"For this reason your case is of high interest, and well worthy of being published *in extenso.* I hope you will let me know when and where it appears.

Very truly yours,

ARTHUR VAN HARLINGEN."

## Figures and Tables

**Figure f1:**